# Correction: Cohort profile: The Bristol IVF Study- A longitudinal study of women, their partners and treatment outcomes following assisted reproductive technologies

**DOI:** 10.1371/journal.pone.0350677

**Published:** 2026-05-29

**Authors:** Amy E. Taylor, Taemi Kawahara, Jennifer Provis, Karema Al Rashid, Sophie Fitzgibbon, Alix Groom, Amanda Jefferys, Paul Wilson, Scott M. Nelson, Valentine Akande, Deborah A. Lawlor

There are errors in the Funding statement. The correct Funding statement is as follows: This study was funded by the European Research Council under the European Union’s Horizon 2020 research and innovation program (grant agreements No 101021566) and the NIHR Biomedical Research Centre at University Hospitals Bristol NHS Foundation Trust and the University of Bristol. The Wellcome Trust and UK Medical Research Council fund the ALSPAC G2 cohort and the clinic facilities that were used in participant follow-up (102215/Z/13/Z). A.T and D.A.L are supported by the UK Medical Research Council (MC_UU_00032/5). The views expressed in this publication are those of the author(s) and not necessarily those of any of the funders, the NHS, or the Department of Health and Social Care. None of the funders influenced the study design or analyses.
